# Investigation of the Strength of Plastic Parts Improved with Selective Induction Heating

**DOI:** 10.3390/polym13244293

**Published:** 2021-12-08

**Authors:** Przemysław Poszwa, Paweł Muszyński, Krzysztof Mrozek, Michał Zielinski, Andrzej Gessner, Michał Kowal

**Affiliations:** 1 Institute of Materials Technology, Poznan University of Technology, Piotrowo 3, 61-138 Poznan, Poland; 2 Institute of Mechanical Technology, Poznan University of Technology, Piotrowo 3, 61-138 Poznan, Poland; pawel.muszynski@put.poznan.pl (P.M.); krzysztof.mrozek@put.poznan.pl (K.M.); andrzej.gessner@put.poznan.pl (A.G.); michal.kowal@put.poznan.pl (M.K.)

**Keywords:** injection molding, elastic hinge, rapid temperature cycling, induction heating

## Abstract

The use of selective induction heating of molding surfaces allows for better filling of molding cavities and has a positive effect on the properties of molded products. This is particularly important in the production of parts that include flexible hinges, which are thin plastic layers connecting two or more parts of the product. By using hinges, it is possible to expand the use of injection molding products and their capabilities. They are widely used in the production of parts for the electrical engineering industry and for packaging Fast Moving Consumer Goods (FMCG). The use of hinges also entails specific reductions in wall thickness. Increases in the shear rate can be expected, which can lead to the degradation of polymers and deterioration of mechanical properties of materials. This paper investigates injection molded flexible hinge parts manufactured with selective induction heating to improve their properties. To verify the efficiency of reduction of material degradation due to high shear rates, open/close tests of elastic hinges were performed. The linear relation between the number of cycles the hinges can withstand, mold temperature and injection time was identified, where mold temperature was the more significant factor.

## 1. Introduction

Increasing demand for high-volume production of low-cost, high-quality products has resulted in increased development of technologies for manufacturing plastic products [1,2,3]. Their main goal is to obtain the best possible product quality while maintaining high repeatability and a low number of defective parts. The duration of the production process is also important as it translates into economic factors. The most popular production method, over the many years it has been in use, in fact, is injection molding [4]. This technology enables the manufacturing of parts that can be used in various industries [5,6,7]. In order to increase the profitability of production, thin-walled products are desired. This is due to the reduced amount of material required and reduced cycle time through faster cooling after injection molding. In thin wall injection molding, there is the problem of incomplete filling of the cavity [8]. This is due to the progressive cooling of the melt while filling the mold cavity. It is a result of the temperature difference between the mold, its forming surface, and the melted plastic in the classical injection mold. Initially, as the temperature decreases, the viscosity and flow resistance of the melt increase [9]. Layers of already frozen polymer also form, which reduce the flow cross section. This phenomenon can result in misruns and product defects. A possibility to overcome this phenomenon is to increase the injection pressure, but this may not be a sufficient countermeasure and it may cause new defects. This problem is especially evident in parts with complex shapes and longer lengths. It is also more common when fillers are used in the material or when materials with higher viscosity are used [10]. This results in defects such as short shots, visible welding lines, diesel effect or warping [11]. It also has a negative influence on mapping of microstructures [12]. Some of these defects can be fixed in additional technological processes. However, for ecological and economic reasons, the aim is to achieve the finished product in a single process.

Rising customer demands for product quality have resulted in the need to address these issues [13,14,15]. Another important parameter of the injection molding process, besides the pressure, is the mold temperature. According to several research results, it is the most important parameter of plastic injection molding [16,17,18]. This has led to the development of Rapid Temperature Cycling (RTC) technology. Its main principle is the cyclic change of the mold temperature during the production process [11,19]. Initially, the mold is heated in order to increase its temperature during the injection of plastic. This is also to ensure better flow and better filling of the molding cavity. After the injection process there follows the cooling stage which lets the material solidify. This process is repeated while being synchronized with technological parameters. In order to properly fill the mold cavity, the temperature of the forming surface should be close to the temperature of the injected melt. The ability to obtain better product quality is achieved by the non-Newtonian nature of plastics. Their rheology is directly related to the processing temperature [20]. The melt viscosity under isobaric conditions is not constant. It changes with the change of shear rate. The RTC technology applied in the injection mold makes it possible to influence the temperature distribution and the course of its changes in the molding cavity. At the same time better pressure distribution in the whole injection molding volume is achieved, i.e., smaller differences between the injection point and far points in the cavity. According to research [21], this allows a reduction in the number of frozen plastic stripes in materials. It also influences the value and direction of shrinkage. Increasing the temperature of the mold, especially its forming surfaces, allows better product quality to be achieved. This positive effect on product surface quality is confirmed by a variety of research results. Better surface quality and better shape reproduction has been achieved in the case of tests presented in the paper [22]. When mapping microstructures, improved results can also be obtained [23,24]. Studies also confirm the improvement of the strength properties of products in which the production process uses methods to increase the mold temperature during injection [25,26,27].

Application of technologies such as steam heating or cartridge heating can cause a significant extension of the cooling phase. This is related to the heating of the whole mold for the injection process. It is also not beneficial from the efficiency point of view of the process. A solution to this problem can be found in new technological solutions such as conformal cooling [28] or by optimizing the heating and cooling process [29]. Heating only the forming surfaces can reduce both the heating time and the subsequent cooling time. One of the technologies that make this possible is heating the forming surfaces with an assisting gas [30,31,32]. This has resulted in improved product surface quality and better filling of the mold cavity, thus defects such as layers of frozen plastic have been eliminated. Huang et al. confirmed that better product quality is obtained by using infrared heating of the molding insert surface [33]. Induction heating technology is characterized by a high heating rate and small heating volume. It allows increasing the temperature during injection molding while not significantly extending the process. Professor Chen and his team carried out work on this topic [34,35]. The main objectives of the work on this technology are to obtain improved control of the heated regions and higher heating rates [36,37,38]. The use of induction heating with the Assistance of a Rotation Device is presented in the paper [39]. This process allowed for a longer melt flow path, an increase from 71.5 to 168.1 mm, and an increase in mold cavity filling from 10.2 to 100%. Confirmation of better surface quality during injection molding of Carbon Fiber Reinforced Semi-Aromatic Polyamide using induction heating was presented in [40]. The effect of induction heating of selected forming surfaces on the strength properties of electrical housing connector was described in the paper [41].

The analysis of the literature revealed that there is no description of selective heating of an injection mold by induction. Therefore, the description of the dependence of the local heating of the mold cavity on the processes occurring inside the flowing polymer is not available. The authors undertook to analyze these phenomena by performing experimental and simulation studies. The first part of the research results was described in study [42]. Strength tests of the samples made by two methods were performed. The first one was the classical injection molding without increasing the mold temperature and the second one was the injection molding with induction heating of selected areas. Application of selective induction heating provided high dynamics of heating process and simultaneously short cooling time. The main focus of the research was the influence of selective induction heating of certain mold areas on the shear rate of the melt. This has a direct effect on the strength properties of the manufactured product. In this publication, emphasis is placed on the verification of the obtained results of simulation tests. The verification was carried out the design and building of the proprietary of the test stand measuring the number of cycles of opening and closing moldings equipped with a flexible hinge.

## 2. Investigated Geometry

A flexible hinge is a thin layer between at least two parts of a molded product with a thickness of 0.15 to 0.5 mm. By using such elements in products, it is possible to extend their range and functionality. During the injection molding, the flow of material through the forming section of the hinge causes the orientation of polymer molecules along the flow path. The next stage of production, which is rapid cooling, maintains this orientation in the part. When a semi-crystalline material is used in the production, the increase in molecular orientation causes the creation of an area with a high degree of crystallinity and high strength. The correct operation of such a hinge is also greatly influenced by its geometry. It must not be too long, thick, or wide. During injection molding, the plastic should flow through the hinge forming part perpendicular to its axis of action.

The presence of a hinge in the part also reduces the flow cross section of the melt flow. This has the effect of reducing the flow rate which depends on the flow resistance and is limited by the volume of the cavity behind the hinge.

**Figure 1:**
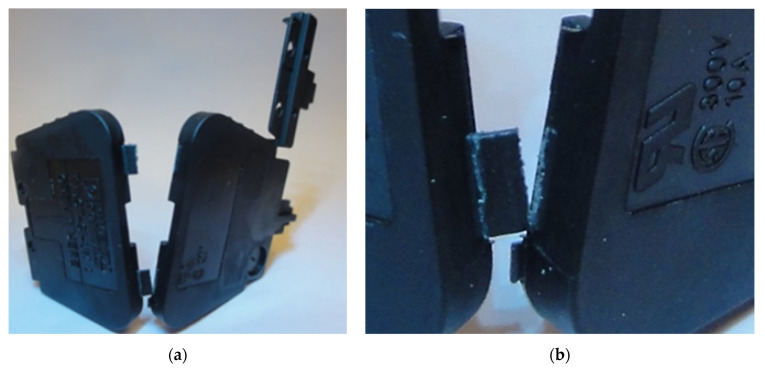
(**a**) Housing of electrical connector made from PA 66 (Frianyl A63 RV0 Frisetta), (**b**) cracking hinges during the part closing process.

To produce high-volume parts with hinges, additional injection points can be used. However, the joining weld line from different points can’t run in the area of the cavity where the hinge is formed. The parameters influencing the durability of the hinge are: type of material used, injection mold design, technological parameters of the process and the shape of the molded part. In the case of amorphous plastics, the durability can be low, the hinges serve only for assembly (closing of the molded part), they are disposable. Hinges for semi-crystalline plastics can have a high durability of up to one million cycles for Polypropylene. A molded part equipped with a flexible hinge which is a housing for an electrical connector is shown in Figure 1. Low durability of such hinges is a big quality problem.

In this study the injection molded parts were manufactured with DEMAG35 injection molding machine. The parts were made of polyamide 6,6 (Frianyl A63 RV0 Frisetta) where variable parameters were: injection time (t_inj_ ∈ {0.45,0.55,0.65,0.75,0.85} [s]) and cavity surface temperature (T_mold_ ∈ {80,130,150,170,190} [°C]).

**Figure 2:**
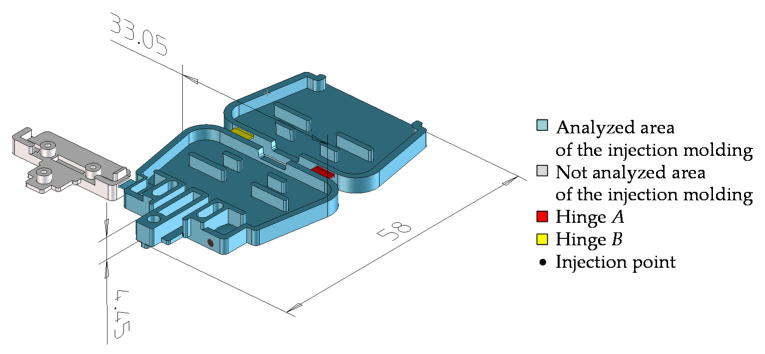
Research model of electrical connector housing.

In order to conduct the tests, a molded part, shown in Figure 2, was designed. Its shape was selected so as to enable better analysis and understanding of the phenomena occurring during the flow of the plastic in the molding cavity. The molding was devoid of micro-features interfering with the formation of the mesh for the purposes of simulation studies. Furthermore, it has been designed in a way to allow a reduction in costs of manufacturing an injection mold, thus allowing the production of products in conventional technology and with selective induction heating.

**Figure 3:**
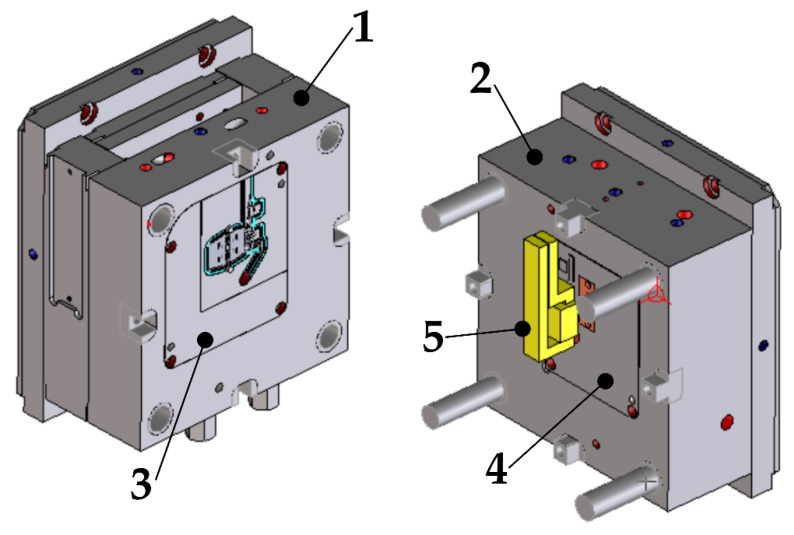
Injection mold model, (1) core part, (2) cavity part, (3) core side forming insert, (4) cavity side forming insert, (5) induction coil.

Figure 3 shows a model of the mold used to make parts presented in Figure 1 and Figure 2. It is equipped with a cold-runner system. The molding inserts (3, 4) located in both parts of the mold are equipped with their own cooling systems. The figure also shows a part of the induction coil (5) used to heat up the molding surface. The mold dimensions are 283 mm × 246 mm × 296 (246) mm, and its ejection stroke is 19 mm.

**Figure 4:**
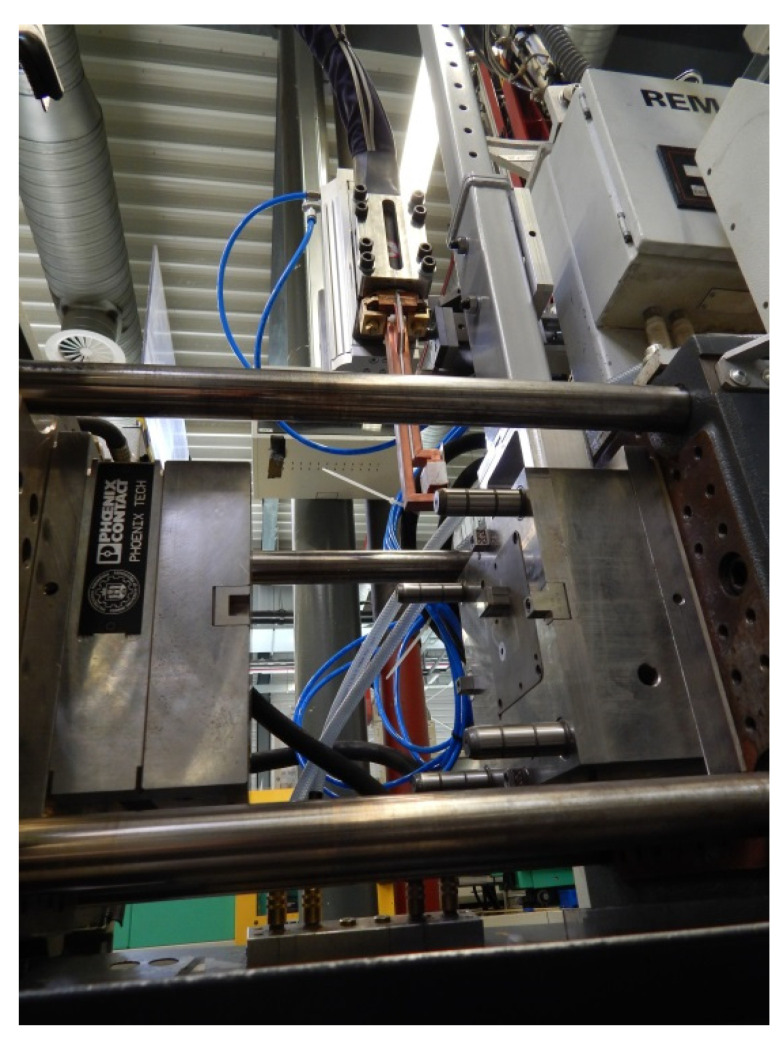
Injection mold.

Figure 4 shows the actual injection mold installed on the injection molding machine during the manufacturing of the test parts along with the visible inductor located on the arm of the pneumatic manipulator.

Using the products made of polymeric materials that are equipped with flexible hinges brings many advantages. The hinge is a thin (0.15–0.5 mm) film between at least two parts of one part. The flow of molten plastic through such a thin area causes a dynamic increase in the shear rate, which in turn affects the degradation of the material and weakening of the strength properties of the finished product.

## 3. Experimental Setup

### 3.1. Selective Induction Heating Simulations

In the present article, simulation studies of selective induction heating of injection molds were conducted. The simulation analysis was carried out by using the Finite Element Method (FEM) implemented in the QuickField 6.3.1 package (Tera Analysis, Svendborg, Denmark). All of the tests were performed in 2D (XY) space in AC Magnetics modules (electromagnetic analysis) to determine the current density on the surface of the metal insert, followed by transient heat transfer (thermal analysis) to determine cavity surface temperature $T_{mold}$ as a function of time.

The analyses included materials presented in Table 1.

A theoretical model was used to conduct simulation studies of the induction heating process of the system. At first, input parameters of the process were selected. The first one was the geometry of the forming cavity which was characterized based on the molded part shown in Figure 1 and Figure 2. The properties of the materials used in the system were also taken into account. The next input parameters are those describing the electric current, i.e., the current frequency *f* [kHz] and electric current *I* [A]. The set of parameters is completed by the heating time *t* [s]. The model used for simulation studies was based on the one presented by Mrozek and Chen [11].

Initially, an electromagnetic analysis was performed. To do this, Maxwell’s equations were used. As a result, the distribution of the electricity and energy in the material was described in terms of the scope of the magnetic field. The equations are given as follows:

(1) $$\nabla \times E=-\frac{\partial B}{\partial t},$$

(2) $$\nabla \times H=J+\frac{\partial D}{\partial t}=J_{s}+J_{e}+\frac{\partial D}{\partial t},$$

(3) ∇·D=ρ,

(4) ∇·B=0,

where: *E*—strength of an electric field; *B*—magnetic induction; *t*—time; *H*—magnetic field intensity; *J*—current density; *D*—electric displacement; *J*_s_—source current vector; and *J*_e_—induction current vector.

**Figure 5:**
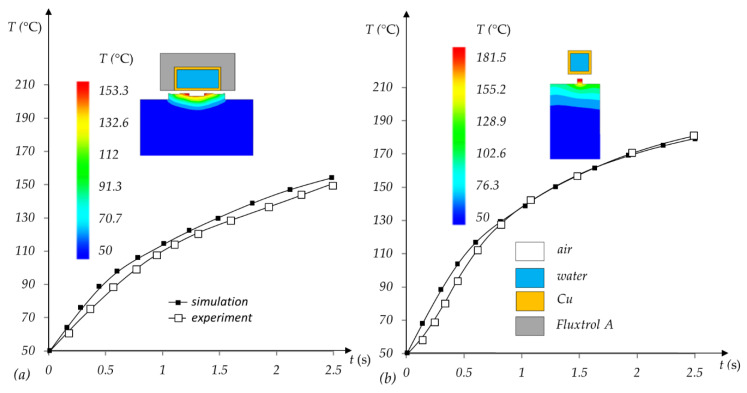
The temperature of the selectively heated region after 2.5 s for the transversely located inductor (**a**) and the longitudinally located inductor (**b**).

The results of the simulation studies have been validated experimentally. For this purpose, tests of induction heating of the cavity insert were carried out in the area of hinge forming, as shown in Figure 2. The induction coil (various variants) is connected to a Minac 6 induction generator. The inductor is placed on a biaxial pneumatic manipulator. When the mold is opened, the coil is moved close to the insert. After heating, the mold closes, and injection begins. The temperature was measured by a FLIR T620 thermal imaging camera (FLIR, Wilsonville, OR, USA), which was calibrated with a Pt100 sensor. The obtained results of simulation and experimental tests are consistent (Figure 5) for both tested solutions—with the coil positioned transversely to the molding insert (Figure 5a) and the coil positioned longitudinally to the insert (Figure 5b).

### 3.2. Injection Molding Simulations

The injection molding simulations were performed with Autodesk Moldflow Insight 2019 software (San Rafael, CA, USA), which uses a Cross-WLF thermorheological model to describe the behavior of polymer melt during the filling of the cavity (it is the material model that is widely used for injection molding simulations [42]).

The complete results of simulations along with experimental setup are present in the previous work [42]. In this paper, the most important numerical results will be presented and compared with experimental results.

### 3.3. Mechanical Tests

**Figure 6:**
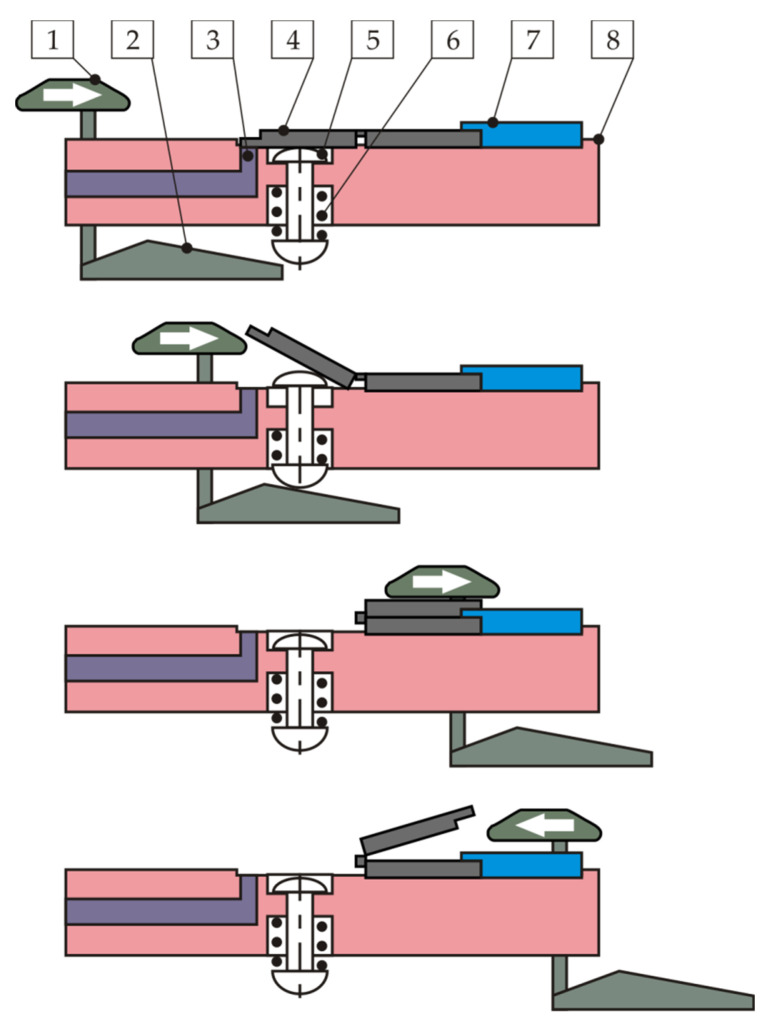
Scheme of the hinge strength test: 1—cam opening/closing the molded part; 2—cam ejecting the pusher; 3—sensor of the part presence; 4—tested molded part; 5—pusher; 6—spring; 7—part clamping; and 8—body of the station.

The strength of the molded hinge was tested independently to measure the number of hinge open and close cycles until fracture. A diagram of the developed concept of opening and closing the molded part is shown in Figure 6. The molded part (4) is placed in the cavity (8) and secured with the clamp (7). Sensors (3) confirm the presence of the movable half of the molded part after each closing cycle. Cams (1 and 2) are connected by a bracket and have the ability to move linearly. Their movement to the right causes the pusher (5) to move, pushing half of the molded part out of the cavity, and then its complete closure by the cam (1). Once the cam assembly has reached its rightmost position, the part begins to open, as a result of movement of the cam (1) to the left, it opens completely. The sensors (3) confirm the presence of the molded part, that means no breakage of the hinge.

**Figure 7:**
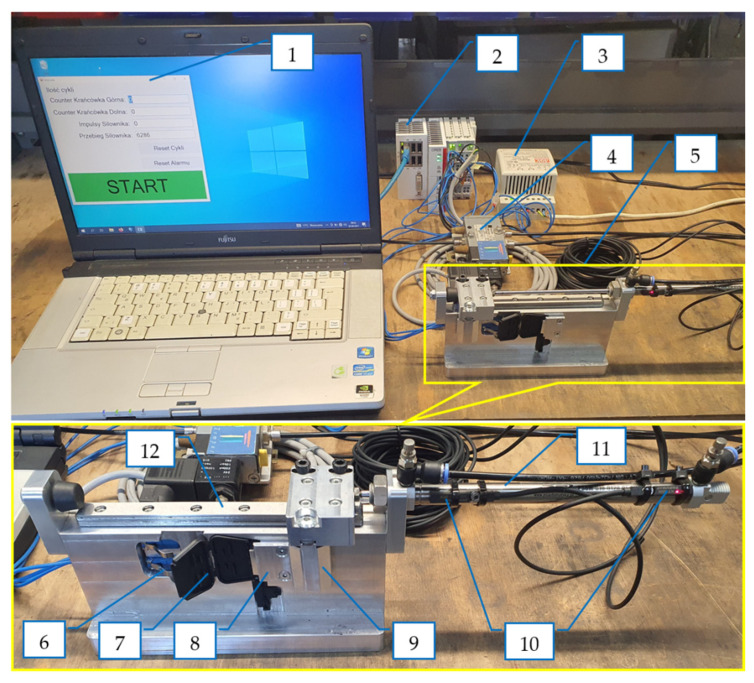
A test stand: 1—computer with data recording software; 2—Beckhoff PLC controller; 3—power supply; 4—pneumatic valve 4/3; 5—molding testing module; 6—molding presence sensors; 7—sample test; 8—clamp; 9—cam opening/closing the molded part; 10—end positions of the pneumatic cylinder; 11—pneumatic cylinder; 12—guide of the cam carriage.

**Figure 8:**
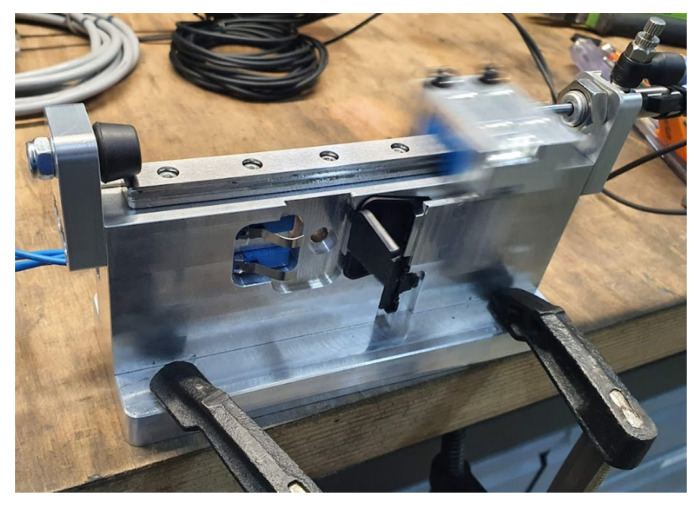
View of the test stand after breakage of the lower hinge.

The view of the test stand built according to the developed concept is shown in Figure 7 and Figure 8. It consists of a computer with data recording software comprised of the following: (1) a Beckhoff PLC controller; (2) a power supply; (3) a 5/2 pneumatic solenoid valve; and (4) a module testing the moldings (5). The tested molding (7) is being cut, placed in the module retaining seat and secured with a clamp (8). The pneumatic cylinder (11) moves the carriage with a cam (9) along the guide (12), which cyclically opens and closes the molding. Two sensors (6) register each case of straightening (opening) the molding, independently for both hinges. In the case of breakage of one of the hinges, the test cycle is carried out further and the software counts the cycles of the remaining openings and closures of the hinge. The PLC controller receives from the sensors (10) confirmation that the extreme positions of the pneumatic cylinder have been reached, corresponding to the opening and closing of the molding. If these positions are not reached within a specified time, the controller indicates a failure and the need for intervention.

## 4. Results and Discussion

The results of the simulation study of selective induction heating of the flexible hinge region are shown in Figure 5. The experimental validation is also shown in this figure. There are also diagrams showing the temperature distribution in the hinge area after a heating time of 2.5 s. Version (a) shows the results for the transversely located inductor and version (b) shows the results for the longitudinally located inductor. For the first solution, a more even temperature distribution in the hinge-forming region was achieved. The maximum *T_mold_* temperature was taken as the temperature measured in the middle of the hinge and its value was 187 °C.

The results from the open/close test were stored in *. xlsx spreadsheet and then the data were visualized with a script prepared in Python language (pandas, SciPy and matplotlib libraries were used) [43,44,45,46,47].

**Figure 9:**
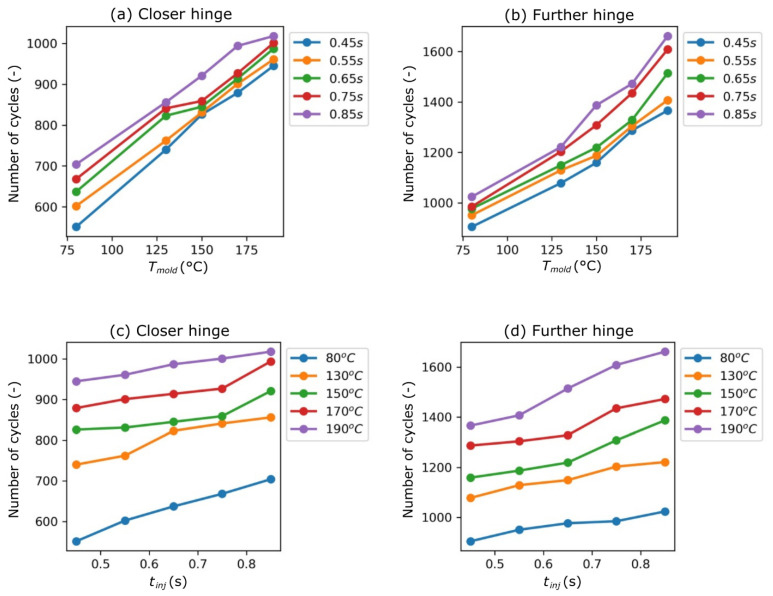
(**a**–**d**) Number of opening/closing cycles of the hinges closer to and further from injection point until fracture.

The results of the hinge open/close tests are presented in Figure 9a–d. For each variant, 5 tests were performed and the table shows the average number of cycles which each hinge can withstand.

The hinge further from the injection point (upper hinge) can withstand more cycles then the other hinge, located closer to the injection point (lower hinge). The difference between hinges comes from different shear rates that occur during filling of the cavities (higher shear rate—smaller number of cycles to withstand. The obtained results show that the strength of the upper hinge is approximately 50% greater than that of the lower hinge, a range of 42% to 68%). Extending the injection time has a positive effect on the strength of the hinges of the molded part. For extending the injection time by 50%, the strength was increased by 10%. It was found that extending the production cycle of the part is unfavorable.

A significant improvement in hinge strength was obtained by increasing the mold temperature as a result of the induction heating method. With regard to the reference temperature of 80 °C, while maintaining the same injection time, the strength of the upper hinges increased by 39–50%, and the lower hinges by 59–71%. By using selective induction heating technology to manufacture these types of parts, it is possible to significantly improve the strength of the hinges.

The next step was to investigate the relation between number of cycles that the hinge can withstand, $t_{inj}$ and $T_{mold}$. For both variables, the relation was linear (for $t_{inj}$ the $R^{2}>0.95$, for $T_{mold}$ the $R^{2}>0.9$ in every case). It was decided to check if it is possible to describe the relation with one equation, so Equation (1) was used:

(5) $$n=a_{1}t_{inj}+a_{2}T_{mold}+a_{3}$$

The ordinary least squares method was to fit the plane described with Equation (5). According to the results, in this case $R^{2}$ was equal to 0.986 for hinge closer to injection point and 0.958 for hinge further from injection point. The coefficients of fitted planes can be found in Table 2.

The linear relation between number of cycles, $t_{inj}$ and $T_{mold}$ is clearly presented in this study. In the previous study [42], the linear relation between maximum shear rate ${\dot{\gamma }}_{max}$ through thickness and $T_{mold}$ was present.

The attempt to correlate the linear relation of ${\dot{\gamma }}_{max}\left(T_{mold}\right)$ and linear relation of $n\left(T_{mold}\right)$ brought about no significant relation. The difficulties came from the fact, that in previous work, the ${\dot{\gamma }}_{max}$ was measured through thickness, and averaging of ${\dot{\gamma }}_{max}$ did not bring any meaningful results.

## 5. Conclusions

This paper investigates the effect of using induction heating on the strength properties of a flexible hinge produced by injection molding. The described model of a molded part with hinges allowed the conducting of a series of tests and analysis. The first stage was to investigate the achievable temperature of the hinge forming region of the insert. For a heating time of 2.5 s, it was possible to achieve a temperature of 187 °C in the middle of the surface. The next step was to study the filling of the molding cavity by the melt in order to determine the shear rate at which degradation of the polymer can occur. In order to analyze their properties, the parts produced for different parameters were subjected to a test that determined the number of opening/closing cycles of the hinges until failure.

The polymer grade used in this study was Frianyl A63 RV0 Frisetta with a maximum shear rate of ${\dot{\gamma }}_{max}$ of 60,000 s^−1^. The application of selective induction heating for $t_{inj}>0.45\;s$ allowed the reduction of the maximum shear rate for both hinges below the critical value. Due to the presented structure of the molded part, both hinges have different mechanical properties, which is confirmed by the conducted tests. This is due to the different ${\dot{\gamma }}_{max}$ value during production. The reason is the difference in local melt viscosity during injection molding. A hinge with a lower shear rate can withstand more cycles in the open/close test. This is related to the specific shear rate of the material; going beyond this range means the degradation of the material and thus a decrease in its strength properties.

Thanks to the obtained results it was possible to analyze the influence of process parameters on the number of opening/closing cycles of flexible hinges. The first parameter whose effect was studied was the injection time. According to the results obtained, increasing the injection time by approximately 50% allows an approximately 10% higher strength of the hinges. The next analyzed parameter was the injection mold temperature. The results obtained for various temperatures during the injection process showed a much bigger influence of this parameter on strength. Increasing the temperature by 80 °C allowed an increase in the number of open/close cycles before damage. For the upper hinge, this was an increase of approximately 45% and for the lower hinge approximately 65%. This significant increase in strength was achieved by using selective induction heating. The comparison of simulation studies with experimental ones presented in the paper illustrates the significant temperature increase achievable in 2.5 s for this method. For the first inductor position (a) this increase was of approximately 100 °C and for the second position (b) approximate 130 °C. The analysis of the obtained results allowed us to determine the relationship between the number of hinge opening/closing cycles and the studied process parameters, i.e., injection time and mold temperature. The obtained equations are of considerable accuracy. Therefore, based on them, it is possible to estimate the strength of the hinges for the given process parameters.

The results indicate that the increase of cavity surface temperature can significantly improve the mechanical performance of elastic hinges where the influence of cavity surface temperature is much more significant than the influence of injection time.

## Figures and Tables

**Table 1:** Thermal and mechanical properties of mold material.

Property	Steel 1.2343
$\mathrm{Relative}\;\mathrm{magnetic}\;\mathrm{permeability}\;\mu_{r}$	55
$\mathrm{Electrical}\;\mathrm{conductivity}\;\sigma \left[\frac{S}{m}\right]$	1 × 10^8^
$\mathrm{Thermal}\;\mathrm{conductivity}\;K\left[\frac{W}{m\;K}\right]$	45
$\mathrm{Density}\;\rho \left[\frac{g}{cm^{3}}\right]$	78
$\mathrm{Specific}\;\mathrm{heat}\;C\;\left[\frac{J}{kg\;K}\right]$	460

**Table 2:** Coefficients of fitted planes for both hinges.

	Closer Hinge	Further Hinge
$a_{1}\left[\frac{1}{s}\right]$	268.60±22.77	500.20±61.51
$a_{2}\left[\frac{1}{{}^{\circ}C}\right]$	3.173± 0.085	4.83±0.24
$a_{3}\left[-\right]$	208.22±19.51	230.49±52.69

## Data Availability

Not applicable.
